# Elastase- and LPS-Exposed Cpa3^Cre/+^ and ST2^-/-^ Mice Develop Unimpaired Obstructive Pulmonary Disease

**DOI:** 10.3389/fimmu.2022.830859

**Published:** 2022-04-13

**Authors:** Eduardo I. Cardenas, Perla A. Alvarado-Vazquez, Erika Mendez-Enriquez, Erik A. Danielsson, Jenny Hallgren

**Affiliations:** ^1^ Department of Medical Biochemistry and Microbiology, Uppsala University, Uppsala, Sweden; ^2^ Division of Lung and Airway Research, Institute of Environmental Medicine, Karolinska Institute, Stockholm, Sweden; ^3^ Department of Surgical Sciences, Uppsala University Hospital, Uppsala, Sweden

**Keywords:** COPD - chronic obstructive pulmonary disease, pulmonary function, airway hyperresposiveness, mast cell (MC), ST2, IL-33

## Abstract

IL-33 and its receptor ST2, as well as mast cells and their mediators, have been implicated in the development of chronic obstructive pulmonary disease (COPD). However, whether mast cells and the ST2 receptor play a critical role in COPD pathophysiology remains unclear. Here, we performed repeated intranasal administrations of porcine pancreatic elastase and LPS for four weeks to study COPD-like disease in wildtype, ST2-deficient, and Cpa3^Cre/+^ mice, which lack mast cells and have a partial reduction in basophils. Alveolar enlargement and changes in spirometry-like parameters, e.g. increased dynamic compliance and decreased expiratory capacity, were evident one day after the final LPS challenge and worsened over time. The elastase/LPS model also induced mild COPD-like airway inflammation, which encompassed a transient increase in lung mast cell progenitors, but not in mature mast cells. While ST2-deficient and Cpa3^Cre/+^ mice developed reduced pulmonary function uninterruptedly, they had a defective inflammatory response. Importantly, both ST2-deficient and Cpa3^Cre/+^ mice had fewer alveolar macrophages, known effector cells in COPD. Elastase/LPS instillation *in vivo* also caused increased bronchiole contraction in precision cut lung slices challenged with methacholine *ex vivo*, which occurred in a mast cell-independent fashion. Taken together, our data suggest that the ST2 receptor and mast cells play a minor role in COPD pathophysiology by sustaining alveolar macrophages.

## Introduction

Chronic obstructive pulmonary disease (COPD) is an inflammatory disease of the airways predominantly caused by long-term tobacco smoking, and characterized by progressive and irreversible airflow obstruction. Lack of effective treatments and an increasing prevalence among aging populations make COPD a major health problem, and one of the main causes of death worldwide (1, 2). COPD diagnosis relies on pulmonary function testing to evaluate specific lung alterations. COPD patients often exhibit alveolar enlargement (i.e., emphysema), which causes an increase in their total lung capacity (TLC). The chronic inflammation associated with COPD damages the elastic properties of the airways, which is detected as an increase in dynamic compliance (C_dyn_). COPD patients also have an overall decrease in their expiratory capacity (measured as forced expiratory volume in 1 s/forced vital capacity: FEV1/FVC) due to the ongoing airway inflammation, mucus hypersecretion, emphysema, and loss of elastic recoil. The development of COPD appears to be mainly driven by neutrophils, macrophages, and CD8^+^ and CD4^+^ (Th1/Th17) T cells (3). However, the inflammatory response associated with COPD involves several other mediators and cell types such as IL-33 and mast cells (MCs) whose contributions to COPD pathophysiology have not been fully elucidated.

IL-33 is an alarmin predominantly released by the airway epithelium in response to infection and inhaled irritants such as pollution and allergens. IL-33 acts *via* the ST2 receptor on immune cells and structural cells to induce activation and production of proinflammatory mediators, leading to cellular recruitment to the lung and expansion of innate and adaptive immune cells (4). The IL-33/ST2 axis has been most studied in the context of asthma, where IL-33 both enhances allergic responses and plays a pivotal role in antigen-independent responses. Interestingly, expression of IL-33 and the ST2 receptor is increased in lung, serum, and plasma samples from COPD patients (5–7). The increase in IL-33 and ST2 correlates with reduced pulmonary function and higher eosinophil counts in blood, and is more frequent in patients with chronic bronchitis or severe COPD (5, 7). Moreover, an IL-33 gene polymorphism is associated with impaired pulmonary function and early onset of COPD (8). In mice, short-term exposure to cigarette smoke upregulates epithelial IL-33 expression, downregulates ST2 on type 2 innate lymphoid cells, and enhances ST2 expression on macrophages and NK cells, thereby amplifying type 1 immune responses toward influenza infection (5). Although cigarette smoke-induced models of COPD are relevant to human disease, they require long-term exposures to induce the alterations in lung function associated with COPD. In contrast, changes in lung function that recapitulate human COPD can be induced rapidly over a few weeks by administering elastase to the lung (9). Interestingly, intratracheal injection of elastase also induces higher levels of lung IL-33 in mice (10). However, IL-33-deficiency enhances murine emphysema caused by intratracheal administrations of elastase or cigarette smoke extract (10).

MCs are rare ST2-expressing tissue-resident immune cells that release proteases and proinflammatory mediators upon activation by IL-33 (11, 12). Moreover, experimental studies suggest that IL-33-induced activation of MCs participates in key features of asthma (13). For example, IL-33 has been shown to enhance antigen-induced bronchoconstriction *via* MC activation (14), and to induce IL-13-dependent airway hyperresponsiveness *in vivo*, possibly *via* MC activation and release of IL-13 (15). However, MCs activated by IL-33 have also been shown to induce regulatory T cell differentiation and suppression of inflammation in a model of Papain-induced allergic airway inflammation (16). Importantly, MCs have also been implicated in smoke-dependent models of COPD. Mice lacking the MC-specific proteases mMCP-6 or Prss31, which are the mouse homologs of human β- and γ-tryptase, respectively, have less airway inflammation and histological evidence of emphysema (17, 18). There is also evidence of MC-involvement in the human disease. COPD patients have a higher proportion of tryptase- and chymase-expressing MCs (MC_TC_) in the lung and increased tryptase activity in sputum and plasma, all of which correlates with reduced pulmonary function and enhanced disease severity (19, 20). MC numbers are increased in lung biopsies from patients with centrilobular emphysema (21) and chronic bronchitis (22), and MC mediators such as histamine and tryptase are elevated in bronchoalveolar lavage fluid (BALF) from long-term cigarette smokers (23). Altogether, these data indicate that MCs and the IL-33/ST2 axis might be relevant to COPD pathophysiology, but whether they contribute to the impaired pulmonary function that defines COPD has not been thoroughly examined.

In the present study, we investigated how the absence of MCs or the ST2 receptor impacted two key features of COPD: airway inflammation and reduced pulmonary function. For this purpose, ST2-deficient and Cpa3^Cre/+^ mice, which lack MCs and have a partial reduction in basophils, were subjected to a model of COPD induced by repeated intranasal administrations of porcine pancreatic elastase (henceforth elastase) and LPS. While ST2-deficient and Cpa3^Cre/+^ mice had a defective inflammatory response, both strains developed COPD-like impaired pulmonary function. In addition, analysis of precision-cut lung slices (PCLS) revealed that MCs were dispensable for the development of airway hyperresponsiveness (AHR) in elastase/LPS-induced COPD-like disease.

## Materials And Methods

### Mice

Cpa3^Cre/+^ (24) and ST2^−/−^ (25) mice on Balb/c background were originally provided by Hans-Reimer Rodewald and Andrew McKenzie, respectively. These strains and Balb/c JBomTac mice were bred and maintained at the Swedish National Veterinary Institute (Uppsala). All experiments were performed in age- and weight-matched female mice, and conducted in accordance with the ethical permit approved by the Uppsala animal ethics committee (5.8.18-05248/2018).

### Elastase-Dependent COPD Models

**Figure 1:**
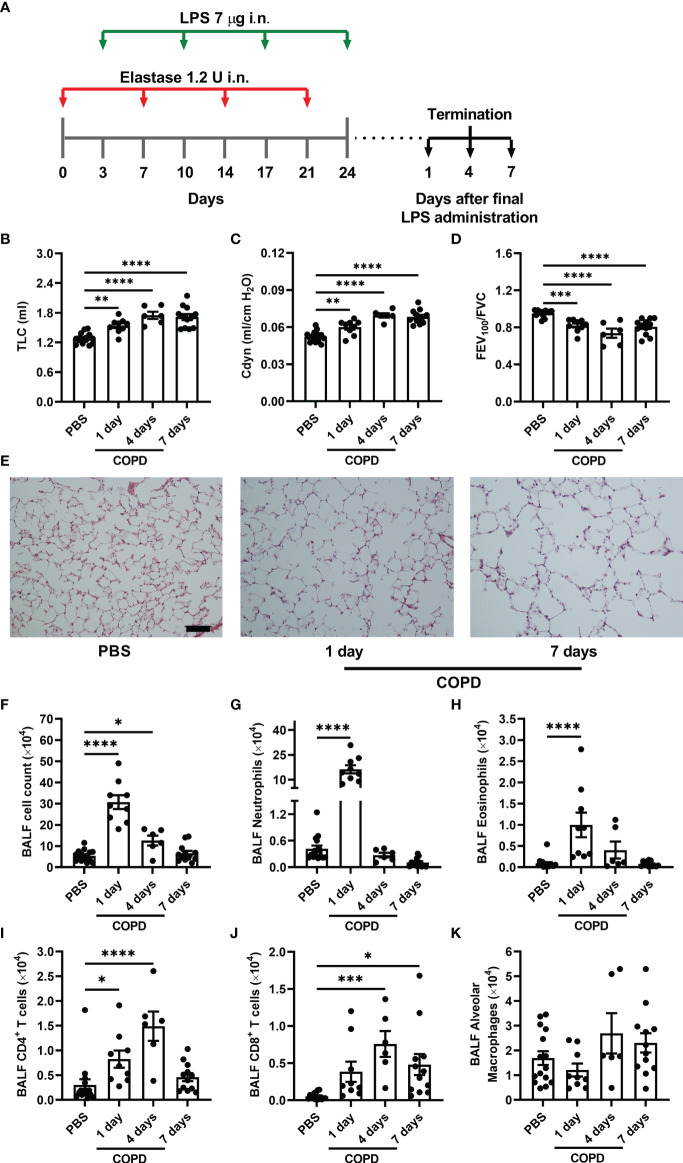
Elastase/LPS instillation causes COPD-like impaired lung function, emphysema, and airway inflammation. **(A)** Wildtype Balb/c mice received alternating intranasal (i.n.) administrations of elastase and LPS for four weeks, and were analyzed one, four or seven days after the final LPS administration. Control mice received PBS instead. **(B)** TLC **(C)** Cdyn, **(D)** FEV_100_/FVC were measured *in vivo*. **(E)** Representative H&E-stained lung sections selected to visualize emphysema development. Scale bar = 200 μm. **(F)** Total cells, **(G)** neutrophils, **(H)** eosinophils, **(I)** CD4^+^ T cells, **(J)** CD8^+^ T cells, and **(K)** alveolar macrophages were quantified in BALF. Data were obtained from 6-15 mice per group pooled from 2-3 individual experiments and shown as means ± SEM. Statistical significance was tested by one-way ANOVA followed by Dunnett’s *post hoc* test to compare each group against the control. *p < 0.05, **p < 0.005, ***p < 0.0005, ****p < 0.0001.

Mice (8 ± 1 weeks old, 20 ± 1 g) received porcine pancreatic elastase (1.2 U in 50 μl phosphate buffer saline [PBS] i.n.) on day 1 and LPS from *Escherichia coli* O26:B6 (7 μg in 50 μl PBS i.n., both from Sigma-Aldrich) on day 4 for four consecutive weeks as shown in **Figure 1A**. Control mice received the same volume of PBS on the indicated days. All administrations were performed under isoflurane anesthesia, and pulmonary function, airway inflammation, emphysema development and AHR were evaluated 1, 4 or 7 days after the final LPS administration. Alternatively, mice received a single dose of elastase (1.2 U in 50 μl PBS i.n.) or PBS (50 μl i.n.) as shown in **Supplementary Figure 2A**, and pulmonary function was evaluated 21 days later.

### Pulmonary Function Testing

Mice were anesthetized (100 mg/kg ketamine, 20 mg/kg xylazine, and 3 mg/kg acepromazine i.p.), tracheotomized, and connected to a Buxco pulmonary function test system (Data Sciences International; DSI). Because pulmonary function measurements are influenced by size (26), only weight-matched mice were compared. We report the average of 3 consecutive measurements for each parameter evaluated.

### Flow Cytometry

To assess airway inflammation by flow cytometry, mice were euthanized and BALF obtained by flushing and aspiration of 1 ml of PBS through a tracheal cannula. Lungs were perfused through the right ventricle (PBS, 10 ml), excised, cut in small pieces, and digested on a gentle MACS Octo Dissociator using a mouse lung digestion kit (both from Miltenyi Biotec). Undigested debris was removed from the lung cell suspension *via* 44% Percoll (Sigma-Aldrich) centrifugation (400 g, 20 min). For residual red blood cell elimination, lung cells were incubated in lysis buffer (1 min; 150 mM NH_4_Cl, 9.5 mM NaHCO_3,_ 1.2 mM EDTA) on ice before extensive washing. Isolated BALF and lung cells were counted in a hemocytometer using trypan-blue exclusion and stained with the antibodies listed in **Supplementary Table 1**. Alveolar macrophages (CD45^+^ Siglec-F^+^ CD11c^+^), eosinophils (CD45^+^ Siglec-F^+^ CD11c^-^), neutrophils (CD45^+^ Siglec-F^-/lo^ CD11c^-/lo^ CD11b^+^ Ly6G^+^), and CD4^+^ (CD45^+^ Siglec-F^-/lo^ CD11c^-/lo^ CD11b^-/lo^ Ly6G^-/lo^ CD3^+^ CD4^+^) and CD8^+^ (CD45^+^ Siglec-F^-/lo^ CD11c^-/lo^ CD11b^-/lo^ Ly6G^-/lo^ CD3^+^ CD8^+^) T cells were identified in BALF as shown in **Supplementary Figure 1**. Mature MCs (CD45^+^ lineage [Lin]^-^ c-kit^hi^ ST2^+^ FcϵRI^+^ CD16/32^+^ Integrin β7^lo^) and MC progenitors (CD45^+^ Lin^-^ c-kit^hi^ ST2^+^ FcϵRI^+^ CD16/32^+^ Integrin β7^hi^) were identified in lung tissue as shown in **Supplementary Figures 3, 4**. Lin was defined as: B220^+^ CD3^+^ CD4^+^ CD8b^+^ Gr-1^+^ TER-119^+^ CD11b^+^. Basophils (CD45^+^ Lin^-^ c-kit^-^ FcϵRI^+^ CD49b^+^) were identified in lung tissue as shown in **Supplementary Figure 4**. Gating of FcεRI^+^ and ST2^+^ cells were set using fluorophore-matched isotype controls. Flow cytometry was performed on a LSRFortessa™, and data was analysed using FlowJo™ (both from BD Biosciences).

### Histology

To qualitatively assess emphysema development, mice were euthanized, and their lungs filled manually using a 2 ml syringe (1.5 ml optimal cutting temperature [OCT] compound). After fixation (4% PFA in PBS), they were cryoprotected (20% sucrose in PBS), and embedded in OCT compound. Cryosections (8 μm) were stained with hematoxylin and eosin (H&E) and imaged with a Nikon Eclipse 90i Upright microscope (Nikon Instruments).

### Preparation of PCLS and AHR Determination

PCLS were prepared as described previously (27). In brief, mice were euthanized, tracheotomized, and their lungs filled with pre-warmed (37°C) low-melting point agarose (4% w/v in PBS; SeaPrep). Whole mice were immersed on ice until agarose solidified, and PCLS (200 μm) were prepared from the left lobe using a vibratome (VT1000 S; Leica Biosystems). Individual PCLS were incubated (37°C, 5% CO_2_) in 1 ml of MEM without phenol red (Gibco), and media was changed every 30 min for the first 2 h, and every h for the next 2 h to remove agarose. Following overnight incubation (37 °C, 5% CO_2_) in 1 ml of RPMI-1640 without FCS (Sigma-Aldrich), individual PCLS were placed in 2 ml MEM and immobilized using a nylon thread attached to a platinum rod. Bronchioles surrounded by a smooth muscle layer (observed as a grey shadow) were time lapse-imaged every 10 s with a Nikon Eclipse Ti2-E inverted microscope. The first min was used as baseline, and the contraction in response to methacholine (100 μM), and subsequently to KCl (60 mM) was recorded for 3 min each. Bronchioles that failed to respond to at least one of these reagents were considered dead and excluded from the final analysis. Bronchial lumen area for each image was calculated using FIJI/ImageJ and changes in lumen area were expressed as fold decrease from baseline. Bronchioles were divided according to their lumen area at baseline into three groups: small (< 5000 μm^2^), medium (5000-15000 μm^2^), and large (15000-40000 μm^2^), and comparisons were performed among similar-sized bronchioles.

### Statistical Analysis

Multiple comparisons were performed by one-way ANOVA followed by Dunnett’s *post hoc* test. Comparisons between two groups were done by two-tailed unpaired Student’s t-test or Mann-Whitney U test (for values that were not normally distributed). All analyses were carried out in GraphPad Prism 9.1.

## Results

### Elastase/LPS-Treated Mice Develop COPD-Like Airway Inflammation and Reduced Pulmonary Function

To induce COPD-like disease, weight- and age-matched female mice received alternating intranasal administrations of elastase and LPS for four weeks (**Figure 1A**). Mice subjected to this model developed larger lung volumes (increased TLC), higher C_dyn_, and decreased expiratory capacity (lower FEV_100_/FVC) than the control mice, which only received PBS (**Figures 1B** **–D**). This impaired pulmonary function was apparent one day after the final LPS administration, worsened over the next days, and was accompanied by histological evidence of alveolar enlargement (i.e. emphysema) (**Figure 1E**).

To characterize the inflammatory populations associated with elastase/LPS-induced COPD-like disease, cells in BALF were analyzed by flow cytometry (**Supplementary Figure 1**). A major increase in inflammatory cells in BALF was found one day after the final LPS administration, which partly resolved over the following days (**Figure 1F**). The inflammatory cells in BALF consisted primarily of neutrophils, accompanied by a smaller increase in eosinophils, one day after the final LPS administration (**Figures 1G, H** **)**. A small peak in the number of CD4^+^ and CD8^+^ T cells was found four days after the final LPS administration (**Figures 1I, J** **)**. The elastase/LPS treatment did not significantly change the number of alveolar macrophages (**Figure 1K**). Altogether, our data suggest that the elastase/LPS model induces a transient inflammation that is not sustained.

### The ST2 Receptor Plays a Minor Role in Elastase/LPS-Induced COPD-Like Airway Inflammation

**Figure 2:**
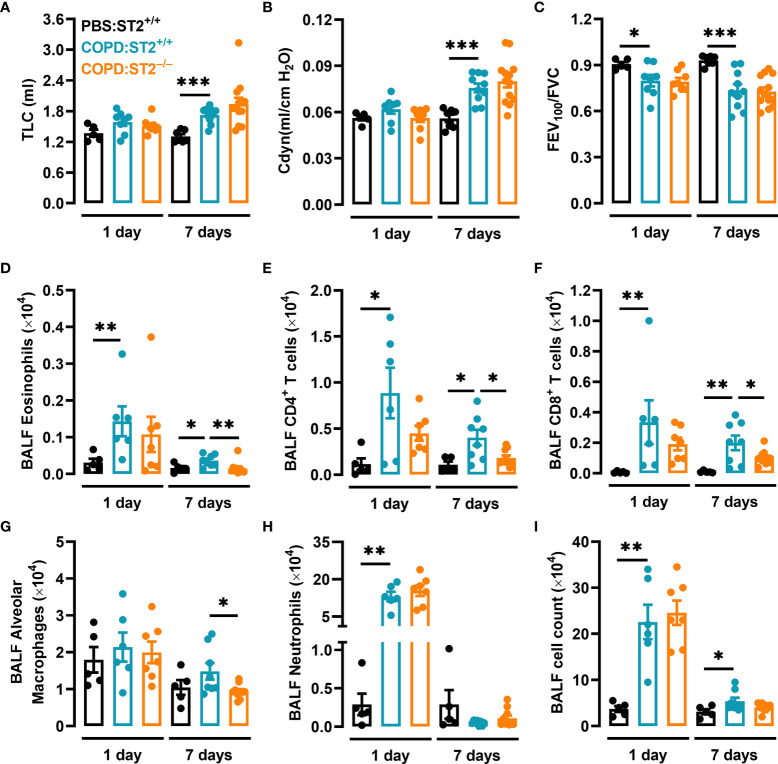
The ST2 receptor is redundant for the development of COPD-like reduced pulmonary function but required for the induction of an intact inflammatory response. ST2^+/+^ and ST2^−/−^ mice were subjected to elastase/LPS-induced COPD-like disease and analyzed one and seven days after the final LPS administration. Control mice received PBS instead. **(A)** TLC, **(B)** Cdyn, and **(C)** FEV_100_/FVC were assessed *in vivo*. **(D)** Eosinophils, **(E)** CD4^+^ T cells, **(F)** CD8^+^ T cells, **(G)** alveolar macrophages, **(H)** neutrophils, and **(I)** total cells were quantified in BALF. For the 1-day time point, data were obtained in **(A-C)** from 5-8 mice per group pooled from 5 individual experiments, and in **(D-I)** from 5-7 mice per group pooled from 4 individual experiments. For the 7-day time point, data were obtained in **(A-C)** from 7-13 mice per group pooled from 5 individual experiments, and in **(D-I)** from 8-10 mice per group pooled from 4 individual experiments. Data is shown as means ± SEM. Statistical significance was tested by Mann-Whitney U test. *p < 0.05, **p < 0.005, ***p < 0.0005.

Given that both clinical and animal studies have implicated the IL-33/ST2 axis in COPD, we hypothesized that the IL-33 receptor ST2 would play a role in elastase/LPS-induced COPD-like disease. However, when ST2-deficient mice (ST2^−/−^) and their wildtype littermates (ST2^+/+^) were subjected to this model, no differences were found between the pulmonary function outcomes (TLC, C_dyn_, and FEV_100_/FVC) of each group, neither at one nor at seven days after the final LPS administration (**Figures 2A** **–C**). Nevertheless, quantification of inflammatory cells in BALF revealed that ST2-deficient mice had fewer eosinophils (**Figure 2D**), CD4^+^ T cells (**Figure 2E**), CD8^+^ T cells (**Figure 2F**), and alveolar macrophages (**Figure 2G**) than ST2^+/+^ mice seven days after the final LPS administration. In contrast, ST2 deficient mice had similar neutrophil numbers and overall BALF cell count as their wildtype littermates (**Figures 2H, I** **)**.

Recently, Morichika et al. used a less severe model of COPD (a single dose of elastase) to show that IL-33-deficiency enhanced emphysema development in mice (10). We adopted this model, and evaluated pulmonary function 21 days later (**Supplementary Figure 2A**). Elastase alone did not induce increased TLC (**Supplementary Figure 2B**) but caused slightly higher C_dyn_ (**Supplementary Figure 2C**) and decreased FEV_100_/FVC (**Supplementary Figure 2D**). Still, ST2^−/−^ and ST2^+/+^ mice developed similar C_dyn_ and FEV_100_/FVC after a single dose of elastase (**Supplementary Figures 2E, F** **)**. To summarize, our data suggest that the ST2 receptor is redundant for the development of elastase- and elastase/LPS-induced COPD-like impaired pulmonary function. Nonetheless, the ST2 receptor is required for an intact elastase/LPS-induced airway inflammation.

### Elastase/LPS-Treated Cpa3^Cre/+^ Mice Have Fewer Alveolar Macrophages

**Figure 3:**
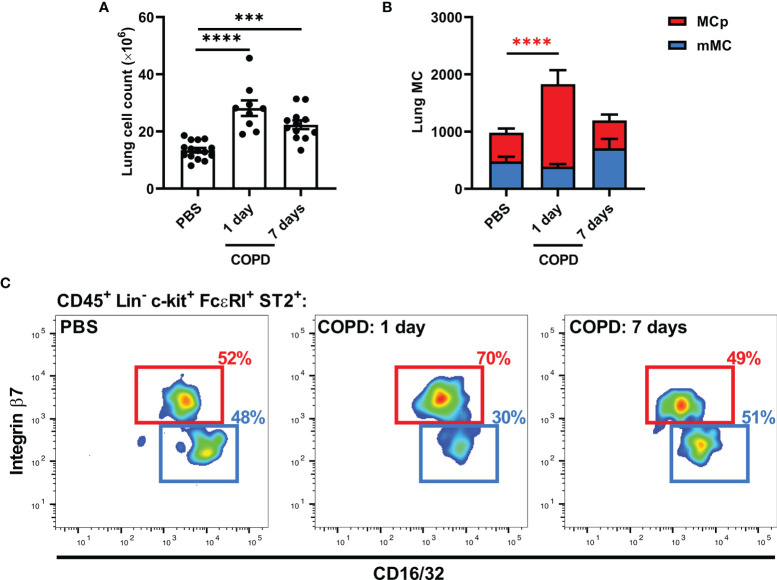
Elastase/LPS instillation induces an increase in lung MC progenitors. **(A-C)** Wildtype Balb/c mice were subjected to elastase/LPS-induced COPD-like disease and analyzed one or seven days after the final LPS administration. **(A)** Total lung cells and **(B)** lung MCs [mature MCs (mMC) and MC progenitors (MCp)] were quantified in lung. **(C)** Representative pseudo-color plots showing the final gates for MCp and mMC, which express different levels of CD16/32 and integrin β7. Data in **(A, B)** were obtained from 9-15 mice per group pooled from 2-3 individual experiments. Data are shown as means ± SEM. Statistical significance was tested in **(A, B)** by one-way ANOVA followed by Dunnett’s *post hoc* test to compare each group against the control. ***p < 0.0005, ****p < 0.0001.

We have previously shown that viral and allergic airway inflammation induce the recruitment of MC progenitors to the lung, which precedes the accumulation of lung MCs (28–30). To determine whether MCs and their progenitors accumulate in elastase/LPS-induced COPD-like disease, cells from dissociated lung tissue were analyzed by flow cytometry (**Supplementary Figure 3**). Elastase/LPS-treated mice had increased total cell numbers in lung tissue one and seven days after the final LPS administration (**Figure 3A**). Although the number of mature MCs did not change, there was a transient 2-fold increase in MC progenitors one day after the final LPS administration that was lost over time (**Figures 3B, C** **)**.

**Figure 4:**
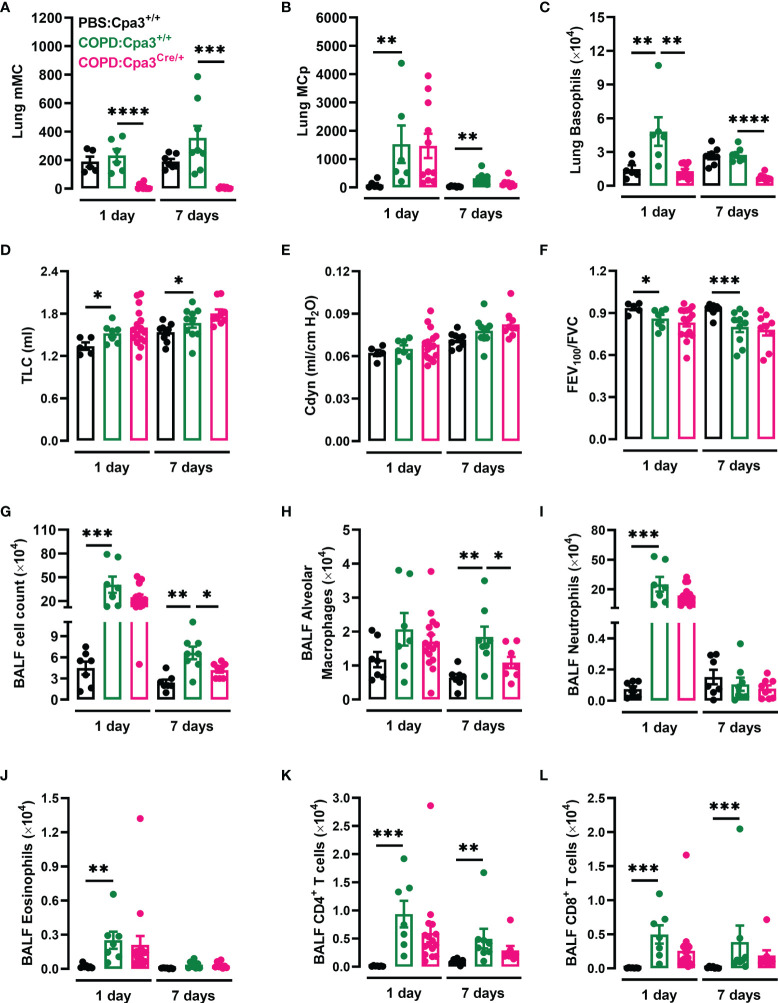
Cpa3^Cre/+^ mice have a defective inflammatory response upon elastase/LPS treatment. Cpa3^+/+^ and Cpa3^Cre/+^ mice were subjected to elastase/LPS-induced COPD-like disease and analyzed one or seven days after the final LPS administration. Control mice received PBS instead. **(A)** Mature MCs (mMC), **(B)** MC progenitors (MCp), and **(C)** basophils were quantified in lung. **(D)** TLC **(E)** Cdyn, and **(F)** FEV_100_/FVC were determined *in vivo*. **(G)** Total cells, **(H)** alveolar macrophages, **(I)** neutrophils, **(J)** eosinophils, **(K)** CD4^+^ T cells, and **(L)** CD8^+^ T cells were quantified in BALF. For the 1-day time point, data in **(A–C)** were obtained from 6-11 mice per group pooled from 3 individual experiments, and in **(D–L)** from 7-16 mice per group pooled from 4 individual experiments. For the 7-day time point, data in **(A–C, G–L)** were obtained from 8 mice per group pooled from 4 individual experiments, and in **(D-F)** from 9-10 mice per group pooled from 5 individual experiments. Statistical significance was tested by Mann-Whitney U test. *p < 0.05, **p < 0.005, ***p < 0.0005, ****p < 0.0001.

To investigate whether MCs play a crucial role in elastase/LPS-induced COPD-like disease, Cpa3^Cre/+^ mice, which lack mature MCs due to the carboxypeptidase A3 (Cpa3)-dependent genotoxic overexpression of Cre recombinase (24), were used to model MC deficiency. Using flow cytometry (**Supplementary Figure 4**), we confirmed that Cpa3^Cre/+^ mice with COPD-like disease lacked mature MCs in the lung (**Figure 4A**). However, they had equal numbers of lung MC progenitors as their wildtype littermates (**Figure 4B**). Similar to what was previously reported in naïve and other disease models (24, 27, 31), Cpa3^Cre/+^ mice with COPD-like disease had a 73% reduction in basophil numbers at both time points (**Figure 4C**). Cpa3^Cre/+^ and Cpa3^+/+^ mice developed similar impaired pulmonary function after elastase/LPS treatments (**Figures 4D** **–F**). Still, Cpa3^Cre/+^ mice had a reduction in total BALF cell count seven days after the final LPS administration (**Figure 4G**), which was at least partly due to a decrease in the number of alveolar macrophages (**Figure 4H**). Nonetheless, BALF neutrophil, eosinophil and T cell numbers were similar in Cpa3^Cre/+^ and Cpa3^+/+^ mice with COPD-like disease (**Figures 4I** **–L**).

Recently, Shibata et al. showed that emphysema development required basophil-derived IL-4 in a single dose of elastase model (32). In our study, Cpa3^Cre/+^ mice were not protected from developing impaired pulmonary function caused by a single dose of elastase (**Supplementary Figures 5A, B** **)**. To conclude, Cpa3^Cre/+^ mice developed unimpaired elastase- and elastase/LPS-induced COPD-like reduced pulmonary function, but had a reduced inflammatory response following elastase/LPS-instillation.

### Elastase/LPS-Treated Mice Develop AHR Independently of MCs

**Figure 5:**
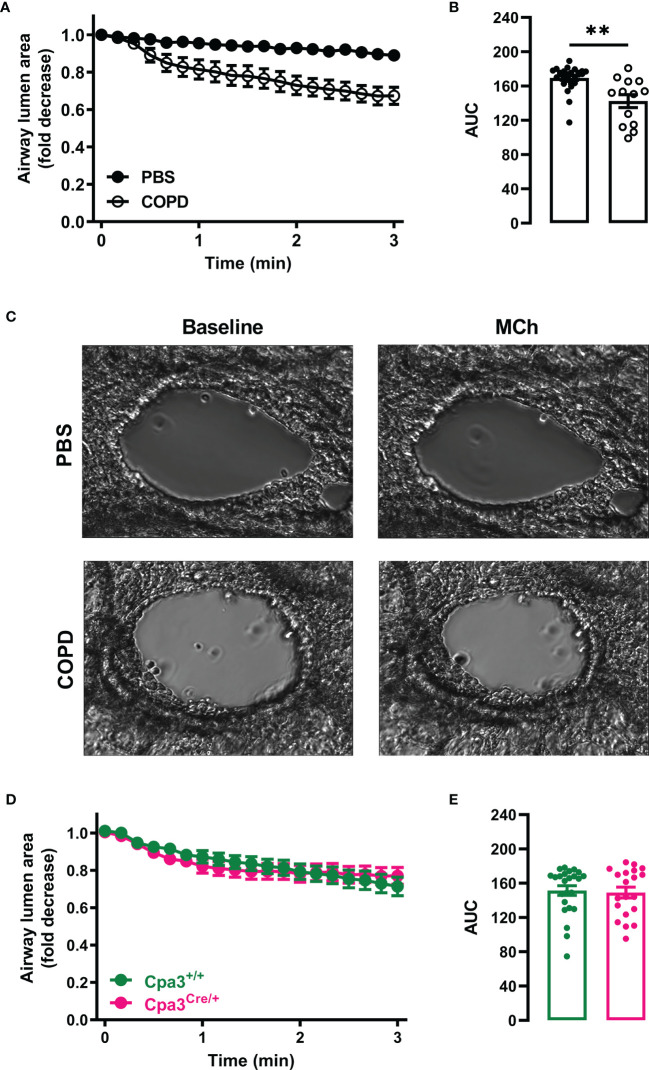
Elastase/LPS instillation causes AHR in large bronchioles independently of MCs. PCLS were obtained from **(A–C)** wildtype Balb/c and **(D, E)** Cpa3^+/+^ and Cpa3^Cre/+^ mice with elastase/LPS-induced COPD-like disease seven days after the final LPS (or PBS) administration. Time-lapse images of individual bronchioles were recorded every 10 s for 1 min before challenge with methacholine (MCh) and recorded for 3 more min. **(A, D)** Airway narrowing after MCh challenge determined as fold decrease from baseline. **(B, E)** Area under the curve (AUC) was calculated for each individual bronchiole. **(C)** Representative images of lung bronchioles before and after MCh challenge. Data in **(A, B, D, E)** are shown as means ± SEM from **(A, B)** 13-27 PCLS per group obtained from 5 PBS and 5 COPD mice pooled from four individual experiments, and **(D, E)** 20-24 PCLS per group obtained from 4 Cpa3^+/+^ and 3 Cpa3^Cre/+^ mice pooled from three individual experiments. Statistical significance in **(B, E)** was tested by unpaired Student’s t-test. ** p < 0.005.

AHR affects one in four patients with mild-to-moderate COPD and is a good indicator of disease progression and mortality (33). To determine whether mice with elastase/LPS-induced COPD-like disease develop AHR, methacholine-induced bronchiole contraction in PCLS was evaluated *ex vivo* seven days after the final LPS administration. Large bronchioles (PBS: 22685 ± 1223 μm^2^, COPD: 24707 ± 1822 μm^2^ lumen area at baseline) from elastase/LPS-treated mice showed stronger contraction (33% ± 0.05 average maximum reduction in airway lumen area from baseline) than those from PBS control mice (**Figures 5A** **–C**). No differences in contraction in response to methacholine were found when analyzing small- and medium-sized bronchioles (**Supplementary Figure 6**). To test whether MCs contributed to AHR in this model, large bronchioles from elastase/LPS-treated Cpa3^+/+^ (22231 ± 1223 μm^2^) and Cpa3^Cre/+^ (21850 ± 1350 μm^2^) mice were analyzed. However, they showed a similar contraction pattern in response to methacholine (**Figures 5D, E** **)**.

## Discussion

The pathological mechanisms behind COPD development are still under investigation. Due to ethical reasons, experimental research on COPD requires animal models. While cigarette smoke-based models are relevant to the human disease, induction of COPD-like disease in mice requires daily cigarette smoke exposure for up to six months, and results in mild non-progressive disease (9). Elastase-instillation, on the other hand, causes severe and progressive emphysema in mice in less than one month (9). By exposing mice to repeated intranasal instillations of elastase and LPS for four weeks, Sajjan et al. established a model in which C57BL/6 mice developed COPD-like airway inflammation and impaired pulmonary function (as determined by a Flexivent system) seven days after the final LPS administration (34). As elastase (10) or LPS alone can induce IL-33 expression in macrophages and epithelial cells (35, 36), we thought that the elastase/LPS model would be relevant to study mechanisms of COPD in mice. In our study, Balb/c mice subjected to the elastase/LPS model developed alveolar enlargement. However, the histological assessment of alveolar enlargement was not quantified and represents a limitation of our study. Instead, emphysema development was quantified by pulmonary function testing, which detected impaired spirometry-like measurements as early as one day after the final LPS administration. Moreover, we confirmed that the elastase/LPS protocol induced mild airway inflammation, with a peak in total BALF cells one day after the last LPS administration, which consisted primarily of neutrophils, followed by a later increase in CD4^+^/CD8^+^ T cells and a gradual decline in total BALF cell numbers.

The IL-33/ST2 axis has been implicated in COPD, and exposure to cigarette smoke or elastase instillation upregulate IL-33 and ST2 in mice (5, 10, 37). However, we did not find a role for the ST2 receptor in the decline in pulmonary function caused by elastase/LPS treatment, nor 21 days after a single elastase instillation. In contrast, IL-33-deficient mice had enhanced static compliance and alveolar enlargement 21 days after a single elastase administration (10). But Morichika et al. also found that antibody-blocking of ST2 could not replicate the increased compliance observed in elastase-treated IL-33-deficient mice (10). Thus, IL-33 may have effects in this setting that are not mediated by the canonical IL-33 receptor ST2.

ST2-deficient mice have impaired type 2 responses, produce less type 2 cytokines (e.g. IL-4 and IL-5) (25), and absence of the ST2 receptor has been shown to impact eosinophil, macrophage, and CD4^+^ and CD8^+^ T cell differentiation (38–41). In agreement with these and other studies that position the IL-33/ST2 axis as key player for the induction of airway inflammation across various settings, ST2-deficient mice subjected to elastase/LPS administrations had fewer BALF eosinophils, alveolar macrophages, and CD4^+^ and CD8^+^ T cells than their wildtype littermates. We speculate that the decline in pulmonary function observed in the elastase/LPS model is largely due to the direct action of elastase alone, and that the mild airway inflammation associated with this model had little impact on pulmonary function. This could potentially explain why the reduced airway inflammation in ST2-deficient mice after elastase/LPS treatment did not lead to an improved pulmonary function. Alternatively, the method used to measure pulmonary function may not be sensitive enough to pick up small differences.

MCs express ST2, and are activated by IL-33 to produce a wide array of mediators that could potentially participate in COPD pathophysiology (42). However, Cpa3^Cre/+^ mice, which lack MCs and have a partial reduction in basophils, developed a similar decline in pulmonary function as their wildtype littermates after elastase alone, or elastase/LPS treatment. Interestingly, Shibata et al. found that diphtheria toxin-injected *Mcpt8^DTR^* mice, which have a complete lack of basophils, developed significantly less elastase-induced emphysema (32). This discrepancy can have (at least) two explanations: 1) MCs are redundant for the development of elastase- and elastase/LPS-induced COPD-like emphysema and the partial reduction in basophils observed in Cpa3^Cre/+^ mice is not enough to recapitulate the findings of Shibata and colleagues, or 2) the absence of MCs in Cpa3^Cre/+^ mice is pathological and compensates for the protection granted by having fewer basophils. On the other hand, Mcpt8 gene expression is not completely restricted to basophils. El Hachem et al. identified Mcpt8 expression in granulocyte-monocyte progenitors, which caused depletion of additional cell lineages such as eosinophils and neutrophils in diphtheria toxin-treated Mcpt8^DTR^ mice (43). Furthermore, Mcpt8-driven Cre-mediated recombination was also detected in <25% of peritoneal MCs, and <10% of other cell lineages in Basoph8 x iDTR mice (44). Future research is needed to dissect the specific contributions of MCs and basophils in COPD.

Since MCs play a major role in AHR in house dust mite-induced allergic airway inflammation (27), we determined whether they could also play a role in AHR in elastase/LPS-induced COPD-like disease. Previously, Van Dijk et al. demonstrated that incubating PCLS from naïve mice with elastase caused AHR *in vitro* (45). In our study, PCLS prepared from mice with elastase/LPS-induced COPD-like disease had increased contraction in response to methacholine *ex vivo* when large bronchioles were assessed. However, Cpa3^Cre/+^ mice developed AHR to methacholine to a similar degree as their wild type littermates. Of note, the average maximum contraction of these bronchioles (33 ± 0.05%) was approximately half of the contraction observed in PCLS from mice with house dust mite-induced allergic airway inflammation (57 ± 5%) (27). It is possible that the mild AHR observed *ex vivo* in PCLS from elastase/LPS-treated mice could explain why Sajjan et al. were unable to detect AHR *in vivo* after elastase/LPS instillation (34). Alternatively, the difference in AHR induction could be due to the use of different mouse strains.

MCs accumulate in the airway smooth muscle of patients with centrilobular emphysema, and those with higher MC number at this location also had increased AHR (21). Other studies found changes in MC distribution and phenotype, and higher sputum tryptase levels that were associated with increased COPD severity (19, 20). In mouse models of allergic airway inflammation, MCs accumulate due to the recruitment and expansion of MC progenitors, which is required for full-blown AHR (27). However, in the current study, elastase/LPS instillation induced only a small increase in MC progenitors, which was not sustained and did not give rise to an increase in mature MCs in the lung. Furthermore, the levels of the MC-specific protease mMCP-1 and trypsin-like activity (as a surrogate marker of MC tryptase) in BALF were similar in mice with elastase/LPS-induced COPD-like disease and PBS controls (results not shown). To conclude, although our data suggest that MCs are dispensable for the development of AHR and COPD-like reduced pulmonary function in elastase/LPS-treated mice, the model failed to recapitulate the MC-related changes that occur in COPD patients. Thus, further studies are needed to find even better mouse models of COPD.

Alveolar macrophages are thought to play a pivotal role in COPD, and disease progression is associated with an increase in macrophages (46). We found that the number of alveolar macrophages in BALF was reduced after elastase/LPS instillation in Cpa3^Cre/+^ mice. This is in line with studies of mice lacking the MC-specific mediators mMCP-6 or Prss31, which had fewer neutrophils and macrophages in BALF following exposure to cigarette smoke (17, 18). Together, these data suggest that MCs promote expansion of alveolar macrophages in COPD-like disease. Mechanistically, this is in line with a recent publication demonstrating that MCs activated *via* IL-33/ST2 release macrophage-attracting factors, including GM-CSF (CSF2), which promote tumor-associated macrophages responsible for tumor cell proliferation and angiogenesis in a preclinical model of gastric cancer (47). Given that GM-CSF is required for the development of monocyte-derived alveolar macrophages (48), we speculate that the decreased alveolar macrophage numbers in Cpa3^Cre/+^ and ST2^−/−^ mice with COPD-like disease may be explained by a loss of ST2-mediated MC release of such factors. However, as basophils are also reduced in Cpa3^Cre/+^ mice, we cannot exclude the involvement of basophils in the induction of alveolar macrophages.

A limitation of our study is that the experiments were performed exclusively in female mice. Male mice (and their airways) grow faster than females, which has challenging consequences. First, it is difficult to breed enough weigh-matched males to start an experiment, and secondly, they continue to grow differently during the experiment, resulting in mice with unequal size at the end of the experiment. Given that differences in size/weight impact lung function outcomes (26), we decided to use female mice for our study. Future research should evaluate potential sex differences.

To conclude, repeated elastase/LPS instillation caused COPD-like impaired pulmonary function, emphysema, and mild AHR in ST2-deficient, Cpa3^Cre/+^, and their wildtype littermates. However, ST2^−/−^ and Cpa3^Cre/+^ mice had a defective inflammatory response in this model, especially regarding alveolar macrophages, which were reduced in both strains. Given that MCs become activated and release pro-inflammatory mediators in response to ST2 stimulation, it seems possible that MCs stimulated *via* IL-33/ST2 contribute to the expansion of alveolar macrophages that occurs in COPD patients. To the best of our knowledge, this is the first time that the roles of MCs and the ST2 receptor were investigated in a mouse model of elastase/LPS-induced COPD-like disease. Our results complement previous findings in cigarette smoke-induced COPD models, and highlight the need for better mouse models.

## Data Availability Statement

The original contributions presented in the study are included in the article/**Supplementary Material**. Further inquiries can be directed to the corresponding author.

## Ethics Statement

The animal study was reviewed and approved by Uppsala animal ethics committee (5.8.18-05248/2018).

## Author Contributions

EC and JH designed the project. EC performed all *in vivo* experiments, lung function analyses, and flow cytometry. PA-V performed the histological analysis. EM-E performed the tryptase assay and supervised the set-up of precision cut lung slice-experiments. ED helped with some of the initial experiments as part of his master degree project. EC analyzed and compiled the collected data, and performed the statistical analyses. JH interpreted and supervised the data and statistical analyses. EC and JH wrote the paper. All authors read, contributed to and approved the final manuscript.

## Funding

The present study was funded by grants to JH by the Swedish Research Council, Swedish Heart-Lung Foundation, Knut and Alice Wallenberg Foundation, Agnes and Mac Rudberg Foundation, Malin and Lennart Philipson Foundation, Apotekare Hedberg Foundation, Konsul ThC Bergh Foundation, Mats Kleberg Foundation, and Axel and Signe Lagerman Foundation; and to EIC by the Gustaf Adolf Johansson Foundation, Agnes and Mac Rudberg Foundation, and P. O. Zetterling Foundation.

## Conflict of Interest

The authors declare that the research was conducted in the absence of any commercial or financial relationships that could be construed as a potential conflict of interest.

## Publisher’s Note

All claims expressed in this article are solely those of the authors and do not necessarily represent those of their affiliated organizations, or those of the publisher, the editors and the reviewers. Any product that may be evaluated in this article, or claim that may be made by its manufacturer, is not guaranteed or endorsed by the publisher.

## Abbreviations

COPD, Chronic obstructive pulmonary disease; Cdyn, Dynamic compliance; FEV_100_, Forced expiratory volume in 100 milli sec; FVC, Forced vital capacity; H&E, Hematoxylin & Eosin stain; LPS, Lipopolysaccaride; Lin, Lineage; OCT, Optimal cutting temperature compound; PBS, Phosphate buffered saline; PCLS, Precision cut lung slices; TLC, Total lung capacity.
