# Supplementary material for: Elastase- and LPS-Exposed Cpa3Cre/+ and ST2-/- Mice Develop Unimpaired Obstructive Pulmonary Disease
Source: Front Immunol. 2022 Apr 13;13:830859. doi: 10.3389/fimmu.2022.830859 (PMC9043106; doi:10.3389/fimmu.2022.830859)
Supplement: Supplementary file 1 [file DataSheet_1.pdf]

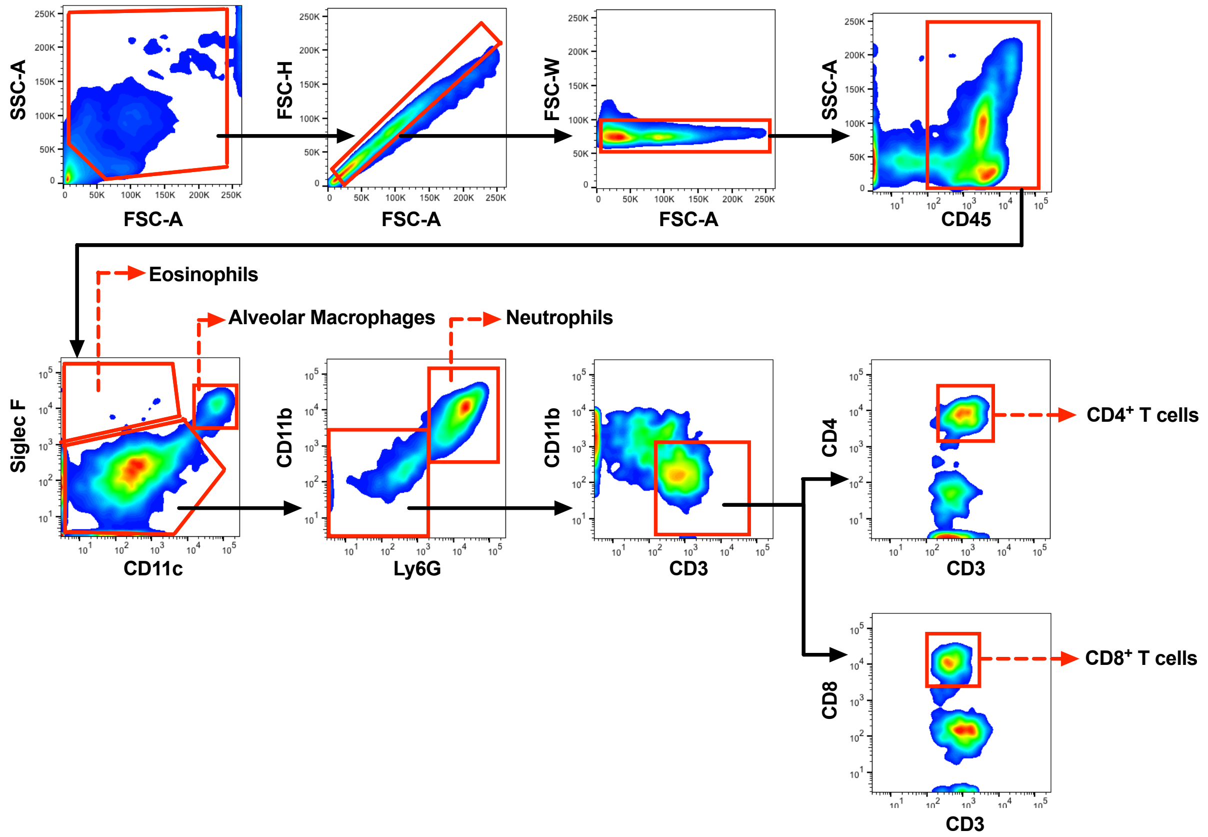

Supplementary Figure 1. Gating strategy for the identification of immune cells in BALF. BALF cells were stained using the antibodies described in Supplementary Table 1 and analyzed by flow cytometry. Alveolar macrophages were gated as $CD45^{+}$ Siglec-F^{+} $CD11c^{+}$ cells, eosinophils as $CD45^{+}$ Siglec-F^{+} $CD11c^{-}$ cells, neutrophils as $CD45^{+}$ Siglec-$F^{-/lo}$ $CD11c^{-/lo}$ $CD11b^{+}$ $Ly6G^{+}$ cells, $CD4^{+}$ T-cells as $CD45^{+}$ Siglec-$F^{-/lo}$ $CD11c^{-/lo}$ $CD11b^{-/lo}$ $Ly6G^{-/lo}$ $CD3^{+}$ $CD4^{+}$ cells, and $CD8^{+}$ T-cells as $CD45^{+}$ Siglec-$F^{-/lo}$ $CD11c^{-/lo}$ $CD11b^{-/lo}$ $Ly6G^{-/lo}$ $CD3^{+}$ $CD8^{+}$ cells.

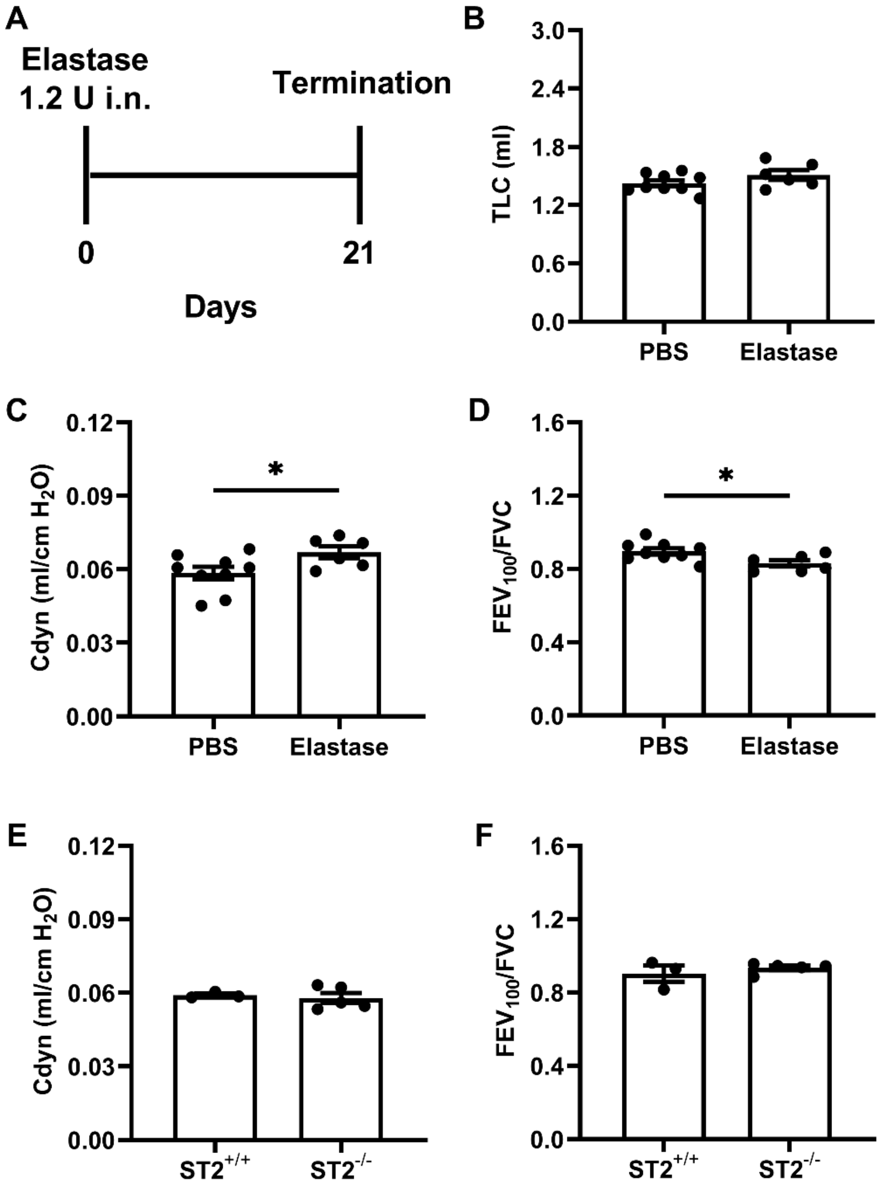

Supplementary Figure 2. A single elastase instillation causes slightly increased Cdyn and decreased FEV_{100}/FVC independently the ST2 receptor. (A) Mice received a single elastase or PBS administration and were analyzed 21 days later. **(B)** TLC, **(C, E)** Cdyn, and **(D, F)** FEV_{100}/FVC were determined. The data are shown as means ± SEM. Data in **(B-D)** were obtained from 6-9 mice per group pooled from 2 individual experiments, and in **(E, F)** from 3-5 mice per group pooled from 2 individual experiments. Statistical significance was tested by unpaired Student's t-test. * $p < 0.05$.

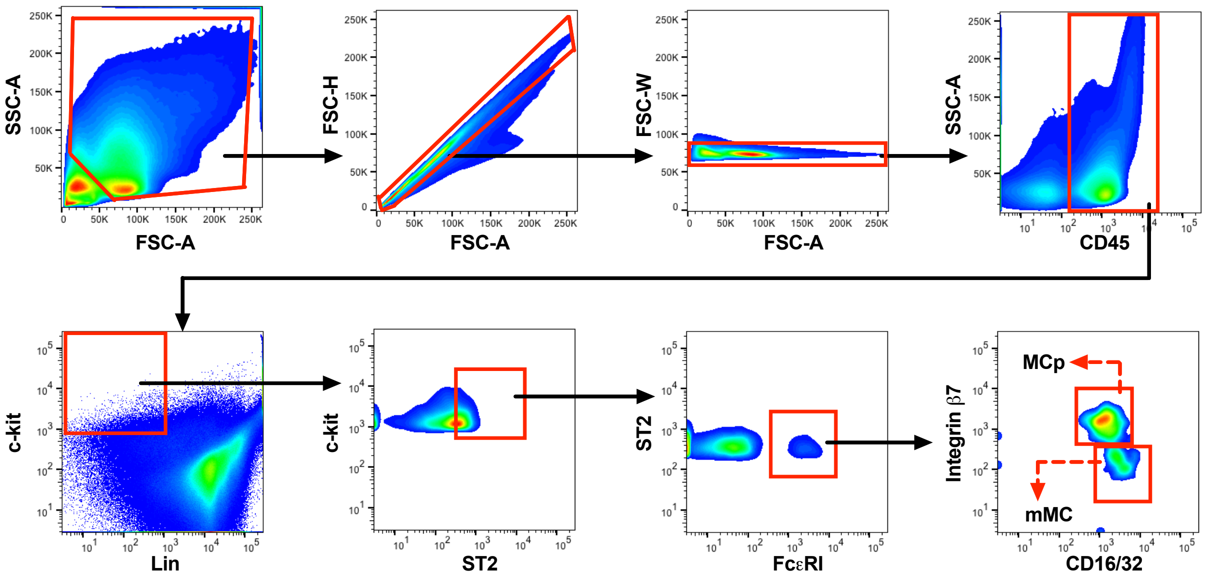

Supplementary Figure 3. Gating strategy for the identification of mature MCs and MC progenitors. Cells from dissociated lungs were stained using the antibodies described in Supplementary Table 1 and analyzed by flow cytometry. Mature MCs (mMC; $CD45^+$ Lin^- c-kit^{hi} $ST2^+$ $Fc\varepsilon RI^+$ $CD16/32^+$ Integrin $\beta 7^{lo}$ cells) and their progenitors (MCp; $CD45^+$ Lin^- c-kit^{hi} $ST2^+$ $Fc\varepsilon RI^+$ $CD16/32^+$ Integrin $\beta 7^{hi}$) were identified.

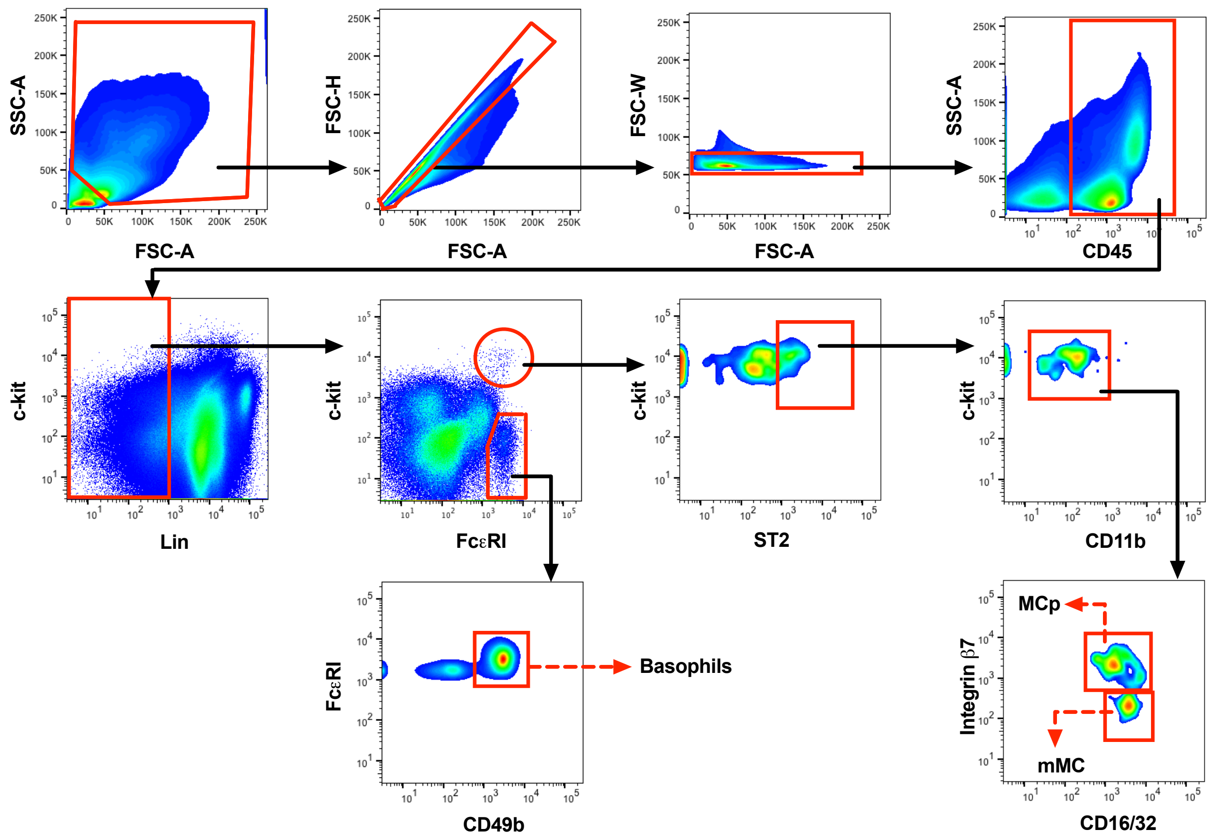

Supplementary Figure 4. Gating strategy for the identification of mature MCs, MC progenitors, and basophils. Cells from dissociated lungs were stained using the antibodies described in Supplementary Table 1, and analyzed by flow cytometry. Mature MCs (mMC) were gated as $CD45^{+}$ Lin^{-} c-kit^{hi} $CD11b^{-}$ $ST2^{+}$ $FcεRI^{+}$ $CD16/32^{+}$ Integrin $β7^{lo}$ cells, MC progenitors (MCp) as $CD45^{+}$ Lin^{-} c-kit^{hi} $CD11b^{-}$ $ST2^{+}$ $FcεRI^{+}$ $CD16/32^{+}$ Integrin $β7^{hi}$ cells, and basophils as $CD45^{+}$ Lin^{-} c-kit^{-} $FcεRI^{+}$ $CD49b^{+}$ cells.

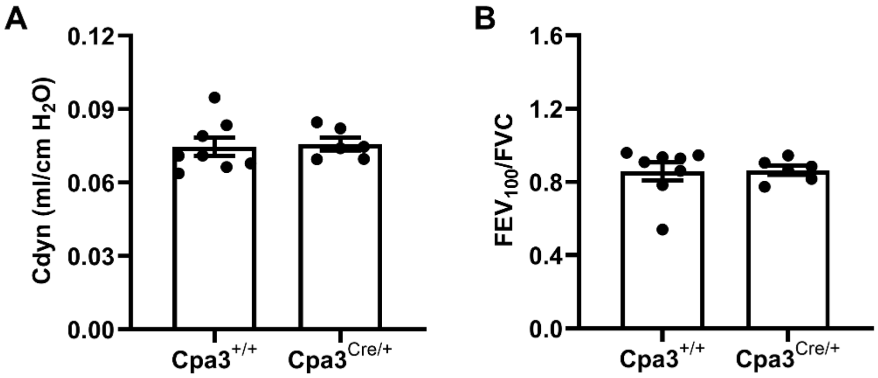

Supplementary Figure 5. A single elastase instillation causes slightly increased Cdyn and decreased FEV_{100}/FVC independently of MCs. Mice received a single elastase administration and were analyzed 21 days later. **(A)** Cdyn and **(B)** FEV_{100}/FVC were determined. The data are shown as means ± SEM. Data were obtained from 6-8 mice per group from a single experiment. Statistical significance was tested by unpaired Student's t-test. No statistically significant differences were found.

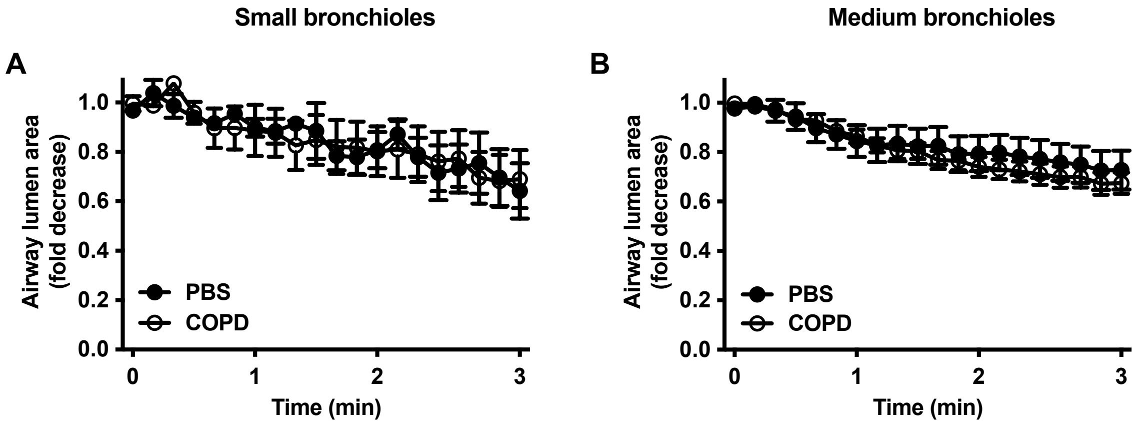

Supplementary Figure 6. Small- and medium-sized bronchioles from elastase/LPS-treated mice do not show AHR in response to methacholine challenge. PCLS were obtained from wildtype Balb/c mice subjected to elastase/LPS-induced COPD-like disease or given PBS as control. Time-lapse images of individual bronchioles were recorded every 10 s for 1 min before **(A)** small- (<5 000 μm^2 airway lumen area) and **(B)** medium-sized (5 000-15 000 μm^2 airway lumen area) bronchioles were challenged with methacholine (MCh) and recorded for 3 more min. Airway narrowing after MCh challenge was determined as fold decrease from baseline. Data are shown as means ± SEM from **(A)** 6-11 PCLS per group obtained from 3 PBS and 5 COPD mice pooled from four individual experiments, and **(B)** 19-37 PCLS per group obtained from 6 PBS and 10 COPD mice pooled from four individual experiments.

Supplementary Table 1. List of antibodies used in flow cytometry.

Supplementary Figure 1			
Target molecule	Fluorochrome	Clone	Company
CD3	PE	17A2	BD Biosciences
CD4	PE-Cy5	GK1.5	Invitrogen
CD8b	BV510	H35-17.2	BD Biosciences
CD11b	FITC	M1/70	BD Biosciences
CD11c	PE-Cy5	N418	Invitrogen
CD45	Alexa Fluor 700	30-F11	Invitrogen
Ly-6G	BV605	1A8	BD Biosciences
Siglec-F	BV421	E50-2440	BD Biosciences
Supplementary Figure 3			
Target molecule	Fluorochrome	Clone	Company
CD45	Alexa Fluor 700	30-F11	Invitrogen
c-kit	PE-Cy7	2B8	Invitrogen
Lin: CD3	PE-Cy5	17A2	BD Biosciences
Lin: CD4	PE-Cy5	GK1.5	Invitrogen
Lin: CD19	PE-Cy5	1D3	Invitrogen
Lin: B220	PE-Cy5	RA3-6B2	Invitrogen
Lin: TER-119	PE-Cy5	TER-119	Invitrogen
Lin: Gr-1	PE-Cy5	RB6-8C5	Invitrogen
Lin: CD8b	PE-Cy5	H35-17.2	Invitrogen
Lin: CD11b	PE-Cy5	M1/70	Invitrogen
ST2	BV421	DIH9	BioLegend
IgG2a (isotype)	BV421	RTK2758	BioLegend
FceRI	PE	MAR-1	Invitrogen
IgG (isotype)	PE	eBio299Arm	eBioscience
CD16/32	BV605	2.4G2	BD Biosciences
Integrin B7	FITC	FIB504	Invitrogen
Supplementary Figure 4			
Target molecule	Fluorochrome	Clone	Company
CD45	Alexa Fluor 700	30-F11	Invitrogen
c-kit	PE-Cy7	2B8	Invitrogen
Lin: CD3	BV510	17A2	BD Biosciences
Lin: CD4	BV510	GK1.5	BD Biosciences
Lin: CD19	BV510	1D3	BD Biosciences
Lin: B220	BV510	RA3-6B2	BD Biosciences
Lin: TER-119	BV510	TER-119	BD Biosciences
Lin: Gr-1	BV510	RB6-8C5	BD Biosciences
Lin: CD8b	BV510	H35-17.2	BD Biosciences
FceRI	PE	MAR-1	Invitrogen
IgG (isotype)	PE	eBio299Arm	eBioscience

**CD11b**	PE-Cy5	M1/70	BD Biosciences
**ST2**	BV786	U29-93	BD Biosciences
**IgG2a (isotype)**	BV786	R35-95	BD Biosciences
**CD16/32**	BV605	2.4G2	BD Biosciences
**Integrin B7**	FITC	FIB504	Invitrogen
**CD49b**	V450	DX5	BD Biosciences
